# O-glycosylation is essential for cell surface expression of the transcobalamin receptor CD320

**DOI:** 10.1016/j.jbc.2024.107997

**Published:** 2024-11-16

**Authors:** Chunyu Du, Wenjun Guo, Mengting Wang, Zibin Zhou, Tiantian Zhou, Meng Liu, Ningzheng Dong, Qingyu Wu

**Affiliations:** 1 NHC Key Laboratory of Thrombosis and Hemostasis, Jiangsu Institute of Hematology, the First Affiliated Hospital of Soochow University, Soochow University Suzhou Medical College, Suzhou, China; 2 Cyrus Tang Hematology Center, Collaborative Innovation Center of Hematology, State Key Laboratory of Radiation Medicine and Prevention, Soochow University, Suzhou, China; 3 Department of Orthopedics, the Second Affiliated Hospital of Soochow University, Suzhou, China

**Keywords:** CD320, cell surface expression, cobalamin, N-glycosylation, O-glycosylation, vitamin B_12_

## Abstract

CD320 is a cell surface receptor that mediates vitamin B_12_ uptake in most tissues. To date, the mechanisms that regulate CD320 expression on the cell surface are not fully understood. In this work, we studied CD320 expression in transfected human embryonic kidney (HEK) 293 and hepatoma HepG2 cells. By glycosidase and trypsin digestion, monensin and brefeldin treatment, western blotting, flow cytometry, and lectin binding, we found that CD320 underwent N- and O-glycosylation and sialylation, resulting in a ∼70-kDa band that formed a high-molecular-weight complex on the cell surface. Site-directed mutagenesis altering Asn126, Asn195, and Asn213 residues, individually or together, abolished N-glycosylation in CD320 but did not block its intracellular trafficking and expression on the cell surface in HEK293 and HepG2 cells. In contrast, treatment of the cells with Ben-gal, a structural analog of GalNAc-α-1-O-Ser/Thr, inhibited O-glycosylation and cell surface expression of CD320 and decreased vitamin B_12_ uptake. Analysis of CD320 deletion mutants indicated that O-glycosylation sites in a Ser/Thr-rich region near the transmembrane domain were important for CD320 expression on the cell surface. These results reveal an important role of O-glycans, but not N-glycans, in the intracellular trafficking and cell surface expression of CD320, providing new insights into the cellular mechanisms in regulating CD320 function and vitamin B_12_ metabolism.

Vitamin B_12_, also called cobalamin, is a water-soluble vitamin critical for normal cellular function (1, 2, 3). As a co-factor for methionine synthase and methylmalonyl coenzyme A (CoA) mutase, vitamin B_12_ participates in nucleic acid, amino acid, and fatty acid synthesis and metabolism. Vitamin B_12_ deficiency, primarily due to insufficient dietary intake or poor intestinal absorption, occurs ∼5 to 10% in people aged 65 years or older (4). Individuals with severe vitamin B_12_ deficiency can develop neurological symptoms (*e.g.*, peripheral neuropathy, gait ataxia, and memory loss) and megaloblastic anemia, a hematological disorder characterized by large and dysfunctional immature red blood cells (1, 2, 5).

Cell surface receptors are essential in vitamin B_12_ metabolism. Cubam- and megalin-mediated endocytosis is crucial in vitamin B_12_ absorption and reabsorption in the intestine and kidney, respectively (1, 6, 7). In most tissues, the uptake of circulating transcobalamin-bound vitamin B_12_ (*i.e.*, holotranscobalamin, holoTC) is carried out by the transcobalamin receptor CD320 (1, 7, 8, 9). Following endocytosis and lysosomal enzyme digestion, vitamin B_12_ freed from holoTC serves its co-factor function in the cytoplasm (for methionine synthase) and mitochondria (for methylmalonyl CoA mutase). The importance of CD320 in vitamin B_12_ metabolism has been demonstrated in genetic studies. In humans, for example, *CD320* variants are associated with high levels of circulating holoTC in an elderly population (10). A *CD320* in-frame deletion variant (p.Glu88del), which impairs holoTC binding and uptake, has been reported in infants with aberrant vitamin B_12_ metabolism (11, 12, 13). In the latest study, autoantibodies targeting CD320 have been identified in patients with neurologic deficits (14). In mice, *Cd320* deficiency decreases tissue vitamin B_12_ levels and impairs peripheral nerve function (15, 16). The mice also develop macrocytic anemia when on a low vitamin B_12_ diet (17). Recently, CD320 expression was identified on the luminal surface of the small intestine and the renal proximal tubules (18). It remains to be determined if CD320 participates in vitamin B_12_ absorption in the gut and reabsorption in the kidney (18, 19).

CD320 is a type I transmembrane protein, consisting of an N-terminal signal peptide, two low-density lipoprotein receptor (LDLR) type A repeats, an epidermal growth factor (EGF)-like module, a transmembrane domain, and a C-terminal cytoplasmic segment (20, 21). The vitamin B_12_ binding is *via* the LDLR repeats, whereas the cytoplasmic tail is critical for holoTC internalization (21, 22). Previously, we identified an amino acid motif, DSSDE, in the second LDLR repeat that functioned as a Rab11a-dependent signal to regulate CD320 expression on the apical membrane in renal and intestinal epithelial cells (18, 23). Disruption of this motif altered the apical expression pattern of CD320 in polarized renal and intestinal epithelial cells. Most likely, additional mechanisms may regulate cellular events in CD320 expression and function.

Glycosylation, particularly N-glycosylation, is a common regulatory mechanism in protein expression and function (24). CD320 is a glycoprotein with three predicted N-glycosylation sites (20, 22). Previous studies with CD320 mutants lacking the transmembrane domain showed that the N-glycosylation sites were not required for holoTC binding (22). In many transmembrane proteins, N-glycans are important determinants in protein folding in the endoplasmic reticulum (ER) and subsequent intracellular trafficking and cell surface expression (25, 26, 27). In this study, we conducted molecular and biochemical experiments in human embryonic kidney and hepatic cells to test if glycosylation plays a role in CD320 expression on the cell surface. Our results may provide new insights into the cellular mechanisms that regulate CD320 biosynthesis and function.

## Results

### Western blotting of CD320 in transfected cells

**Figure 1:**
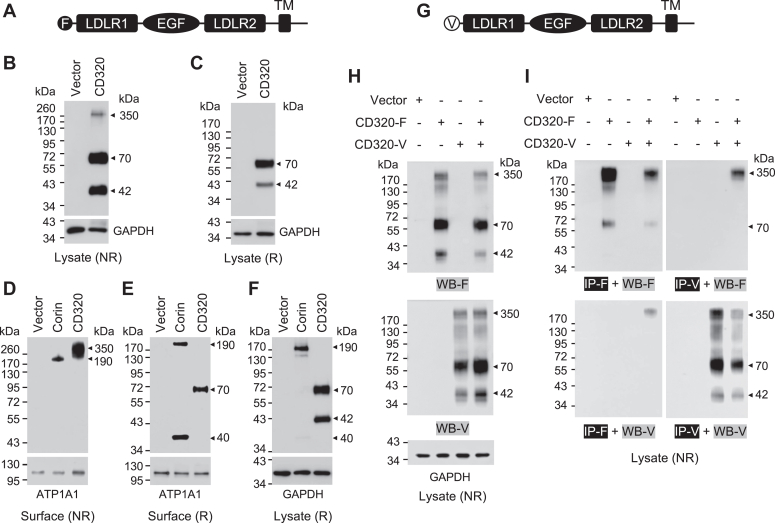
**Western blotting of human CD320 in transfected HEK293 cells.***A*, illustration of CD320 protein modules. F, FLAG tag; TM, transmembrane domain. The signal peptide is not shown. *B* and *C*, western blotting of CD320 in lysates from HEK293 cells transfected with a vector or plasmid expressing human CD320. The blotting was done under non-reducing (NR) (*B*) and reducing (R) (*C*) conditions. GAPDH was a protein-loading control. *D*–*F*, western blotting of corin (control) and CD320 proteins in biotin-labeled cell surface proteins under NR (*D*) and R (*E*) conditions. A Na^+^/K^+^ ATPase 1 (ATP1A1) was a control for membrane proteins. Corin and CD320 protein expression in cell lysates from the same samples were verified by western blotting under R conditions (*F*). *G*, Illustration of CD320 with an N-terminal V5 (V) tag. *H*, HEK293 cells were transfected with a vector or plasmids expressing CD320 with a FLAG (CD320-F) or V5 (CD320-V) tag. Western blotting was done using anti-FLAG (WB-F) or V5 (WB-V) antibodies. *I*, Immunoprecipitation (IP) was done using the anti-FLAG (IP-F) or V5 (IP-V) antibodies followed by western blotting using anti-FLAG or V5 antibodies. Data are representative of at least three experiments.

CD320 is a type I transmembrane protein with a signal peptide, two LDLR-like repeats flanking an EGF-like domain, a transmembrane domain, and a cytoplasmic segment (Fig. 1*A*). We expressed human CD320 with an N-terminal FLAG tag (Fig. 1*A*) in human embryonic kidney 293 (HEK293) cells. By western blotting of cell lysates under non-reducing conditions, we detected two major bands of ∼70 and ∼42 kDa, respectively (Fig. 1*B*). There was another band of ∼350 kDa at a relatively low level (Fig. 1*B*). As a control, no bands were detected in lysates from vector-transfected HEK293 cells (Fig. 1*B*). When the blotting was done under reducing conditions, only the ∼70- and ∼42-kDa bands, but not the ∼350-kDa band, were detected (Fig. 1*C*).

To examine if the detected CD320 bands were on the cell surface, we biotin-labeled cell surface proteins and analyzed them with western blotting. In these experiments, we included a control protein, corin (a transmembrane protease expressed on the cell surface) (28, 29). Consistently, among biotin-labeled surface proteins from corin-expressing cells, a single band of ∼190 kDa (Fig. 1*D*) or two bands of ∼190 and ∼40 kDa (Fig. 1*E*) were detected under non-reducing and reducing conditions, respectively. These two bands represented the full-length corin and the activation-cleaved protease domain band, as reported previously (28). Among biotin-labeled surface proteins from CD320-expressing cells, we detected the ∼350-kDa band under non-reducing conditions (Fig. 1*D*) and the ∼70-kDa band under reducing conditions (Fig. 1*E*). As a control, corin and CD320 proteins were verified in lysates from the same cell samples. A major corin band of ∼190 kDa and two CD320 bands of ∼70 and ∼42 kDa were detected under reducing conditions (Fig. 1*F*). These results indicated that intracellular CD320 existed primarily as two bands of ∼70 and ∼42 kDa, respectively, and that only the ∼70-kDa band was on the cell surface in a complex form.

To verify our findings in different cell types, we conducted similar experiments on CD320 in human hepatoma HepG2 cells. In western blotting of lysates from transfected HepG2 cells, we observed the ∼350-, ∼70-, and ∼42-kDa bands under non-reducing conditions (Fig. S1*A*), and the ∼70- and ∼42-kDa bands under reducing conditions (Fig. S1*B*). We also detected the ∼350- and ∼70-kDa bands under non-reducing and reducing conditions, respectively, among biotin-labeled cell surface proteins (Fig. S1, *C*–*E*). These results indicated that similar CD320 proteoforms were expressed in human kidney- and liver-derived cells.

### Analysis of the CD320 complex form

To understand if the ∼350-kDa CD320 complex form was a homopolymer, we made another plasmid encoding human CD320 with an N-terminal V5 tag (Fig. 1*G*) and expressed CD320 proteins with the FLAG and V5 tags, separately or together, in HEK293 cells. In western blotting of cell lysates under non-reducing conditions, antibodies against FLAG and V5 detected CD320 bands of ∼350, ∼70, and ∼42 kDa with the corresponding tags (Fig. 1*H*). We next did immunoprecipitation using antibodies against FLAG or V5 and analyzed the proteins that were pulled down. In western blotting under non-reducing conditions, the ∼350-kDa V5-tagged CD320 band was detected among the proteins pulled down by the FLAG-tagged CD320 (Fig. 1*I*). Conversely, the ∼350-kDa FLAG-tagged CD320 band was detected among the proteins pulled down by the V5-tagged CD320 (Fig. 1*I*). These results indicated that the ∼350-kDa CD320 complex was likely a homopolymer.

### Effects of trypsin and monensin treatment on CD320 expression

**Figure 2:**
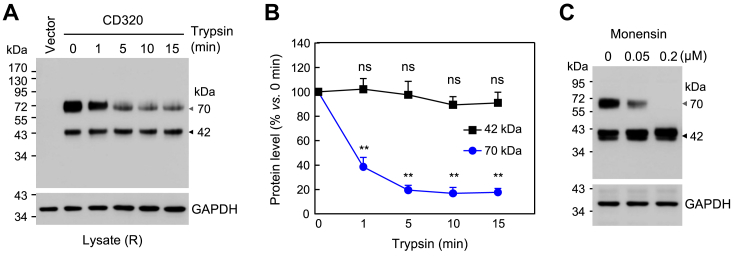
**Analysis of CD320 bands in HEK293 cells treated with trypsin or monensin.***A*, vector- or CD320-expressing plasmid transfected HEK293 cells were treated with trypsin at 37 °C. After the indicated times, the cells were lysed. Western blotting of CD320 in the lysates was done using an anti-FLAG antibody under reducing (R) conditions. GAPDH was a protein-loading control. *B*, Levels of the ∼70- and ∼42-kDa CD320 bands on western blots were quantified by densitometry. Relative protein levels in percentages vs. the levels at 0 min were calculated. Data were mean ± SEM analyzed by one-way ANOVA. ns, not significant; ∗∗*p* < 0.001 vs. 0 min n = 4. *C*, western blotting of lysates from transfected HEK293 cells expressing CD320 that were treated with increasing doses of monensin. GAPDH was a protein-loading control. Data are representative of four experiments.

To distinguish intracellular from cell surface CD320 proteoforms, we treated CD320 expressing HEK293 cells with trypsin to remove cell surface proteins. The trypsin treatment was terminated at different times, and the cells were lysed for western blotting under reducing conditions. Reduced levels of the ∼70-kDa band were observed in the trypsin-treated cells (Fig. 2*A*). Based on quantitative analysis of the protein bands on western blots, levels of the ∼70-kDa band were reduced by ∼80% after 5 min of trypsin treatment. There was no apparent reduction with further trypsin incubation (Fig. 2*B*). In contrast, there was no obvious reduction in levels of the ∼42-kDa band with trypsin digestion (Fig. 2, *A* and *B*). These results indicated that the majority of the ∼70-kDa proteoform was on the cell surface, whereas the ∼42-kDa proteoform was intracellular.

We next treated the CD320-expressing cells with monensin, which disrupts the Golgi network, thereby blocking protein trafficking to the cell surface. As shown in western blotting, levels of the ∼70-kDa band, but not the ∼42-kDa band, were reduced dose-dependently in the monensin-treated cells (Fig. 2*C*). These results indicated that the ∼42-kDa proteoform was likely in the ER, whereas the ∼70-kDa proteoform was likely in the Golgi.

### Analysis of N-glycosylation in CD320

**Figure 3:**
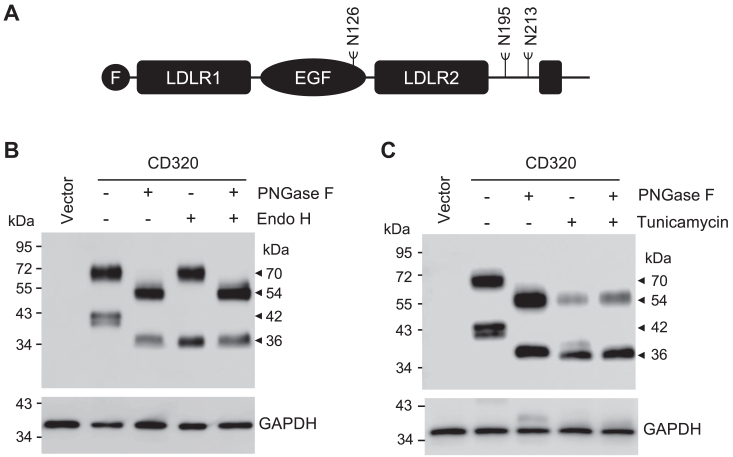
**Effects of glycosidase and tunicamycin treatments on CD320 bands.***A*, illustration of predicted N-glycosylation sites in human CD320. *B*, western blotting of CD320 proteoforms in PNGase F- and/or Endo H-treated lysates from HEK293 cells transfected with a vector or CD320-expressing plasmid. GAPDH was a protein-loading control. *C*, lysates were prepared from HEK293 cells transfected with a vector or CD320-expressing plasmid. The samples were treated with (+) PNGase F and/or (+) tunicamycin or control buffer (−) before western blotting using an anti-FLAG tag antibody. Western blotting was done under reducing conditions. Data are representative of three experiments.

Human CD320 has three predicted N-glycosylation sites at N126, N195, and N213 (Fig. 3*A*). To analyze N-glycosylation in CD320, we treated the lysates from CD320-expressing cells with peptide N-glycosidase F (PNGase F), which removes nearly all N-glycans, and endoglycosidase H (Endo H), which removes high-mannose and hybrid, but not the complex, type N-glycans. As shown in western blotting, PNGase F treatment reduced the ∼70- and ∼42-kDa bands to ∼54 and ∼36 kDa, respectively (Fig. 3*B*). Endo H treatment reduced the ∼42-kDa band to ∼36 kDa but did not alter the mobility of the ∼70-kDa band (Fig. 3*B*). When the lysates were treated with PNGase F and Endo H, no further reduction in the apparent molecular masses of the ∼54- and ∼36-kDa bands (Fig. 3*B*). These results indicated that both the ∼70- and ∼42-kDa CD320 proteoforms were N-glycosylated and that N-glycans on the ∼70-kDa proteoform were the complex type, whereas N-glycans on the ∼42-kDa proteoform were the high-mannose and/or hybrid types.

To verify these results, we treated CD320-expressing HEK293 cells with tunicamycin, an analog of UDP-GlcNAc that blocks N-glycosylation in the ER. In western blotting with lysates from tunicamycin-treated cells, CD320 bands of ∼54 and ∼36 kDa were detected (Fig. 3*C*). These bands migrated at similar positions to those of CD320 bands in PNGase F-treated lysates. In lysates from the tunicamycin-treated cells, PNGase F digestion did not further increase the mobility of the CD320 bands. These results were consistent, indicating that both the ∼70- and ∼42-kDa CD320 proteoforms were N-glycosylated.

### Verification of N-glycosylation at the predicted sites

**Figure 4:**
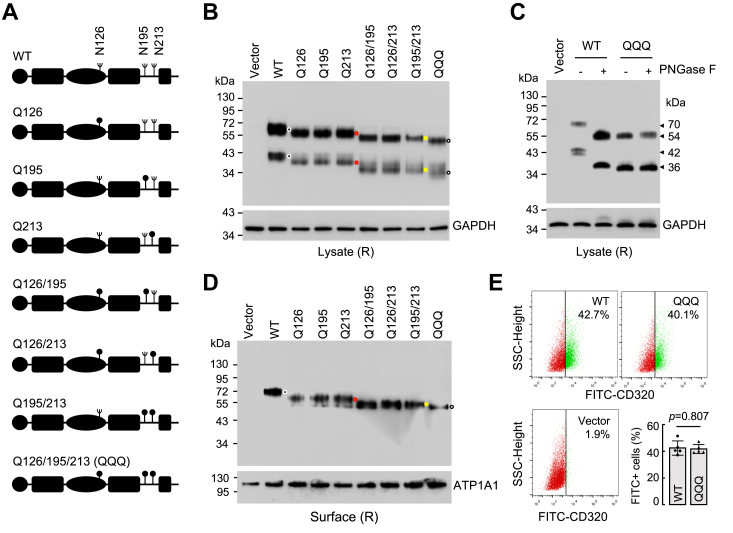
**Effects of N-glycosylation at the predicted sites on CD320 expression in HEK293 cells.***A*, illustration of the predicted N-glycosylation sites in human CD320 and mutants in which N126, N195, and N213 were replaced by Gln (Q) (*dots*), individually or in combination. *B*, western blotting of CD320 proteoforms in lysates from transfected HEK293 cells under reducing conditions (R). CD320 WT appeared as two bands of ∼70- and ∼42 kDa (*black dots*). The mobility of the bands increased progressively in the mutants lacking one (*red dots*), two (*yellow dots*), or three (*white dots*) N-glycosylation sites. *C*, western blotting of buffer (−) or PNGase F (+) treated lysates from HEK293 cells expressing CD320 WT or the mutant QQQ. *D*, western blotting of CD320 proteoforms in biotin-labeled surface proteins from HEK293 cells expressing CD320 WT (*black dot*) and the mutants lacking one (*red dot*), two (*yellow dot*), or three (*white dot*) N-glycosylation sites. ATP1A1 was a control for membrane proteins. *E*, flow cytometric analysis of CD320-positive HEK293 cells transfected with plasmids expressing CD320 WT and the mutant QQQ or a control vector. Data in (*B*–*D*) are representative from three experiments. Data in (*E*) are mean ± SD analyzed by Student’s *t* test. n = 5.

N-glycosylation occurs at the sequon of Asn-X-Ser/Thr, where X can be any amino acid but Pro (30). Local conformation in individual proteins may sterically hinder N-glycan occupancy and maturation (31, 32). To verify N-glycosylation at the predicted sites in CD320, we made mutants in which Asn residues at the positions 126, 195, and 213 were replaced by Gln, individually or in combination (Fig. 4*A*). We expressed the CD320 mutants in HEK293 cells and analyzed the proteins by western blotting. Increased mobilities of the protein bands, corresponding to the ∼70- and ∼42-kDa proteoforms of wild-type (WT) CD320, were observed when one (mutants Q126, Q195, and Q213), two (mutants Q126/195, Q126/213, and Q195/213), or three (mutant Q126/195/213, QQQ) of the N-glycosylation sites were mutated (Fig. 4*B*). When CD320 WT and the mutant QQQ were treated with PNGase F, the ∼70- and ∼42-kDa bands in WT were reduced to ∼54 and ∼36 kDa, respectively. Such reductions were not observed in the mutant QQQ (Fig. 4*C*). These results indicated that all the predicted sites were N-glycosylated and that there were no additional N-glycans on CD320 outside of these sites.

### Effect of N-glycosylation on CD320 cell surface expression

To test if N-glycans are required for CD320 expression on the cell surface, we biotin-labeled proteins on the surface of HEK293 cells. By western blotting, we detected CD320 WT and all the N-glycosylation site mutants among the biotin-labeled cell surface proteins (Fig. 4*D*), indicating that abolishing the N-glycosylation sites, individually or in combination, did not prevent CD320 expression on the surface in the transfected cells. We next quantified the cell surface expression of CD320 by flow cytometry. Similar percentages of CD320-positive cells were observed in HEK293 cells expressing CD320 WT and the mutant QQQ (Fig. 4*E*). To verify these results, we conducted parallel experiments in HepG2 cells. Similarly increased mobilities of the CD320 proteoforms, corresponding to the ∼70- and ∼42-kDa bands in WT, were observed in the N-glycosylation site mutants (Fig. S2*A*). The ∼70-kDa proteoform in WT and the corresponding proteoforms in the mutants were detected among biotin-labeled cell surface proteins by western blotting (Fig. S2*B*). In flow cytometry, percentages of CD320-positive cells were comparable in HepG2 cells expressing CD320 WT and the mutant QQQ (Fig. S2*C*).

### Analysis of O-glycosylation in CD320

**Figure 5:**
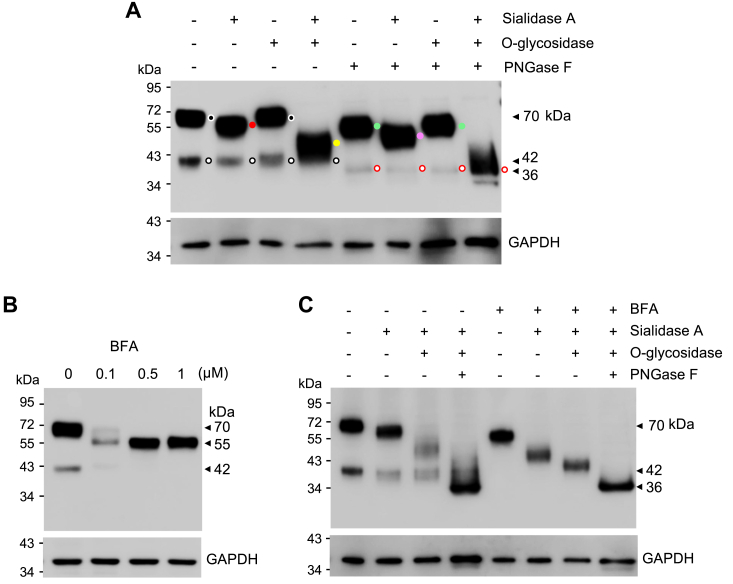
**Analysis of O-glycosylation in human CD320.***A*, CD320 WT was expressed in HEK293 cells. Cell lysates were treated with sialidase A and O-glycosidase, individually or together, without (−) or with (+) PNGase F, followed by western blotting using an anti-FLAG antibody under reducing conditions. CD320 bands of ∼72- (*black dots*), ∼55- (*red dot*), ∼54- (*green dots*), ∼50- (*pink dot*), ∼48- (*yellow dot*), ∼42- (*black lined white dots*), and ∼36- (*red lined white dots*) kDa are indicted. *B*, western blotting of CD320 bands in HEK293 cells cultured without (−) or with increasing concentrations of BFA. *C*, lysates were prepared from CD320-expressing HEK293 cells cultured without (−) or with (+) BFA. The samples were treated with sialidase A, O-glycosidase, and PNGase F, individually or in combination. CD320 proteoforms were analyzed by western blotting under reducing conditions. GAPDH was a protein loading control. Data are representative of three experiments.

In western blotting, the ∼42-kDa band likely represented the N-glycosylated CD320 in the ER, as compared to the theoretical molecular mass (∼29 kDa) of the recombinant human CD320. Upon PNGase F digestion or tunicamycin treatment, the ∼70-kDa band was reduced to ∼54 kDa under reducing conditions (Fig. 3*C*), an indication of additional posttranslational modification(s) in CD320. Based on bioinformatic analysis, there were at least 23 potential O-glycosylation sites in human CD320, most of which were located between the LDLR2 and transmembrane domains (Table S1). To assess potential O-glycosylation on CD320, we treated lysates from HEK293 cells expressing CD320 with sialidase A and O-glycosidase, alone or together, without and with PNGase F. In western blotting, we found that the ∼70-kDa band was reduced to ∼50 kDa after sialidase A treatment (Fig. 5*A*). Such a reduction was not observed in samples treated with O-glycosidase alone (Fig. 5*A*). When the lysates were treated with both sialidase A and O-glycosidase, the ∼70-kDa band was reduced to ∼48 kDa (Fig. 5*A*). In contrast, no apparent reduction was observed in the ∼42-kDa band after sialidase A and O-glycosidase treatment (Fig. 5*A*). When the lysates were treated with PNGase F alone or with sialidase A or O-glycosidase, the ∼70-kDa band was reduced to ∼54, ∼50, and ∼54 kDa, respectively (Fig. 5*A*). When the lysates were treated with PNGase F, sialidase A, and O-glycosidase together, the ∼70-kDa band was reduced to ∼36 kDa (Fig. 5*A*). These results indicated that the ∼70-kDa CD320 proteoform contained N-glycans and terminally sialylated O-glycans, whereas the ∼42-kDa band contained primarily N-glycans.

### Effects of BFA on CD320 glycosylation

Microtubule-mediated retrograde transport of Golgi membrane constituents into the ER is an important cellular mechanism in membrane recycling (33). Brefeldin (BFA), a fungus-derived product, inhibits the anterograde (from the ER to the Golgi), but not the retrograde, pathway in protein transport (33, 34, 35). In HepG2 cells, BFA treatment causes redistribution of glycosyltransferases from the Golgi to the ER (36). Given the finding of O-glycans on CD320, we tested the effects of BFA on CD320 expression. In western blotting of lysates from BFA-treated HEK293 cells, we observed a major band of ∼55 kDa (Fig. 5*B*). When the BFA-treated cell lysates were incubated with sialidase A alone or with O-glycosidase, the size of this band was decreased progressively. When the lysates were treated with sialidase A, O-glycosidase, and PNGase F, the band was reduced to ∼36 kDa (Fig. 5*C*). In control cells without BFA treatment, the ∼70- and ∼42-kDa bands were observed. After the treatment with sialidase A, O-glycosidase, and PNGase F, these two bands were all reduced to ∼36 kDa (Fig. 5*C*). These results indicated that in the presence of BFA that blocked the anterograde protein transport, CD320 may undergo N- and O-glycosylation in the ER.

### Effects of benzyl N-acetyl-α-D-galactosaminide (Ben-gal) on CD320 expression

**Figure 6:**
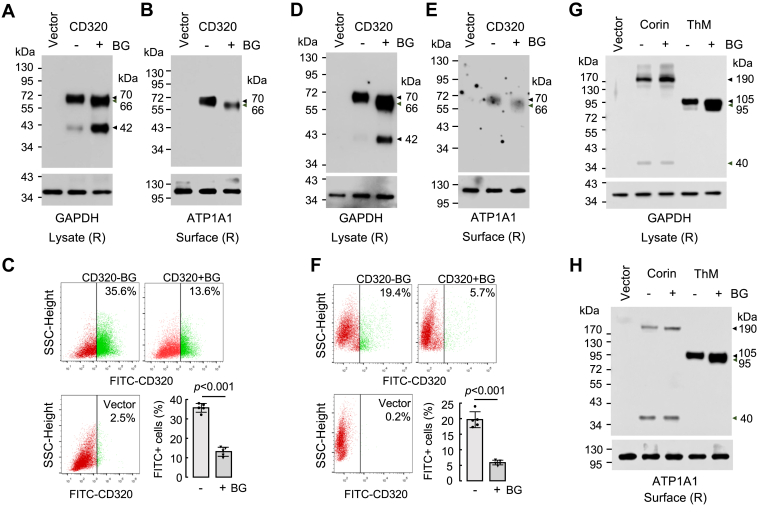
**Effects of Ben-gal treatment on O-glycosylation and cell surface expression of CD320.***A*–*F*, human CD320 was expressed in HEK293 (*A*–*C*) and HepG2 (*D*–*F*) cells cultured without (−) or with (+) Ben-gal (BG) (8 mM). Vector-transfected cells were included as controls. CD320 proteoforms in cell lysates (*A* and *D*) were analyzed by western blotting using an anti-FLAG antibody under reducing (R) conditions. GAPDH was a protein-loading control. *B* and *E*, western blotting of CD320 in biotin-labeled surface proteins from CD320-expressing HEK293 (*B*) and HepG2 (*E*) cells cultured without or with BG. ATP1A1 was a control for membrane proteins. *C* and *F*, Flow cytometric analysis of CD320-positive cells on the surface of vector- or CD320 plasmid-transfected HEK293 (*C*) and HepG2 (*F*) cells cultured without or with BG. *G* and *H*, western blotting of corin and thrombomodulin (ThM) in lysates (*G*) and biotin-labeled surface proteins (*H*) from HEK293 cells transfected with a vector or corin and ThM expressing plasmids was done using anti-FLAG and V5 antibodies. Data in (*A*, *B*, *D*, *E*, *G*, and *H*) are representative of three experiments. Data in (*C* and *F*) are mean ± SD from five experiments analyzed by Student’s *t* test.

Ben-gal is a structural analog of GalNAc-α-1-O-Ser/Thr that inhibits the elongation of O-glycans by acting as a competitive substrate for galactosyltransferase and sialyltransferase activities (37, 38, 39, 40). We tested the effect of Ben-gal on CD320 expression. In western blotting of lysates from Ben-gal-treated HEK293 and HepG2 cells, the ∼70-kDa band was reduced to ∼66 kDa, whereas the intensity of the ∼42-kDa band was increased, compared to those in the untreated cells (Fig. 6, *A* and *B*). Reduced levels of the ∼66-kDa band were also observed in biotin-labeled cell surface proteins from the Ben-gal-treated cells, compared to those of the ∼70-kDa band from the control cells (Fig. 6, *B* and *E*). In flow cytometry, percentages of CD320-positive cells were decreased in the Ben-gal-treated HEK293 and HepG2 cells, compared to those in the control cells (Fig. 6, *C* and *F*). To exclude the possibility that the reduced cell surface CD320 expression was due to increased ectodomain shedding, we examined CD320 proteoforms in the conditioned media from the Ben-gal-treated HEK293 and HepG2 cells. We found that levels of a CD320 band of ∼310 kDa were also lower than those in the conditioned media from the control cells (Fig. S3, *A* and *B*). These results indicated that inhibition of O-glycosylation by Ben-gal impaired CD320 intracellular trafficking and cell surface expression.

In principle, the effect of Ben-gal on the cell surface expression of CD320 could be caused by secondary effects of Ben-gal on intracellular trafficking and surface expression of different proteins with or without O-glycans. To exclude such a possibility, we tested the effect of Ben-gal on the expression corin without detectable O-glycans (41) and thrombomodulin with O-glycans (42) (Table S2). In western blotting of cell lysates and biotin-labeled cell surface proteins, similar corin expression and migration patterns were observed in HEK293 cells with or without Ben-gal treatment (Fig. 6, *G* and *H*). In contrast, the thrombomodulin band in the lysates from Ben-gal treated cells migrated faster than the band from the control cells, indicating that Ben-gal inhibited O-glycosylation in thrombomodulin (Fig. 6*G*). However, levels of the biotin-labeled cell surface thrombomodulin in the Ben-gal treated cells were not reduced, compared to those of the control cells (Fig. 6*H*). These results indicated that the effect of Ben-gal on the cell surface expression of CD320 was unlikely caused by global secondary effects in the cells.

### Analyses of CD320 proteoforms in Ben-gal treated cells

**Figure 7:**
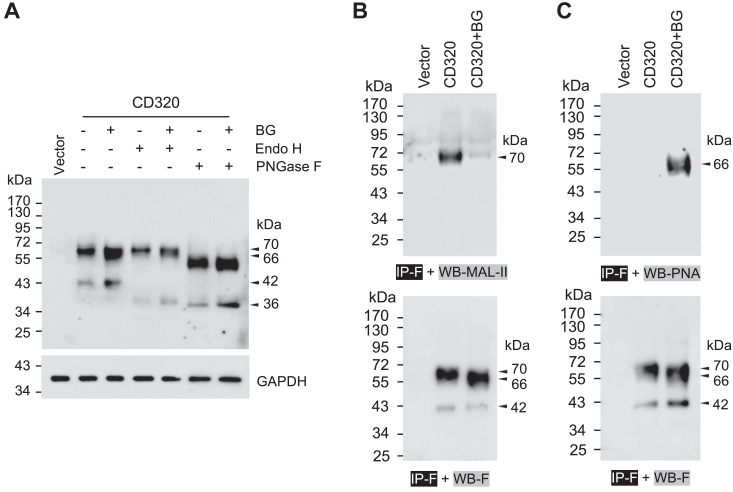
**Analyses of CD320 proteoforms from Ben-gal-treated cells.***A*, effects of Endo H and PNGase F on CD320 proteoforms from Ben-gal-treated cells. HEK293 cells transfected with a plasmid expressing WT CD320 were incubated without (−) or with (+) Ben-gal (BG). After 24 h at 37 °C, the cells were lysed and the lysates were treated with Endo H and PNGase F, alone or together. Western blotting was done under reducing conditions to assess the migration patterns of CD320 proteoforms. *B* and *C*, Lectin binding to CD320 proteoforms from BG-treated cells. WT CD320 was expressed in HEK293 cells in the absence or the presence of BG. CD320 proteoforms were immunoprecipitated with an anti-FLAG antibody (IP-F) and analyzed by western blotting using an anti-FLAG antibody (WB-F) (*lower panels*). The western blots were also probed with labeled maackia amurensis-II (MAL-II) (IP-F + WB-MAL-II) or peanut agglutinin (PNA) (IP-F + WB-PNA) (*top panels*). The data are representative of three experiments.

To test if the Beg-gal treatment caused the retrograde transport of CD320, we examined the sensitivity of the CD320 proteoforms to endoglycosidae H (Endo H). In western blotting of CD320 proteoforms from HEK293 cells without or with Ben-gal treatment, we found that the ∼70- and ∼66-kDa bands were Endo H-resistant, whereas the ∼42-kDa band was Endo H-sensitive (Fig. 7*A*). The results indicated that the ∼70- and ∼66-kDa proteoforms were in the Golgi, whereas the ∼42-kDa proteroform was in the ER, suggesting that Ben-gal reduced the intracellular trafficking of CD320 but did not cause the reverse transport of CD320 from the Golgi to the ER.

We next did lectin binding experiments to assess the effect of Ben-gal on CD320 O-glycosylation. We immunoprecipitated CD320 proteoforms from HEK293 cells without or with Ben-gal treatment and examined the binding of maackia amurensis-II (MAL-II), which binds to α2-3-sialylated Galβ1-3GalNAc in O-glycans, and peanut agglutinin (PNA), which binds to Galβ1-3GalNAc in core 1 O-glycans (43). We found that MAL-II and PNA bound differentially to the ∼70-kDa and ∼66-kDa CD320 proteoforms, respectively, in cells without and with Ben-gal treatment (Fig. 7, *B* and *C*). No binding of MAL-II or PNA to the ∼42-kDa CD320 proteoform was observed. These results indicated that Beg-gal inhibited α2,3-sialylation, but not core one galactosylation, in CD320 O-glycosylation, consistent with previous reports in other O-glycoproteins (44, 45).

### Importance of CD320 N- and O-glycosylation in vitamin B_12_ uptake

**Figure 8:**
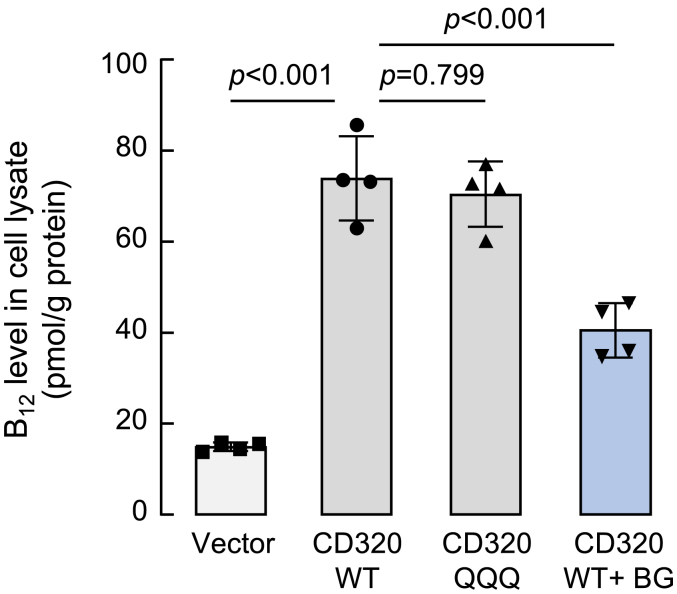
**Vitamin B**_**12**_**uptake in HepG2 cells.** HepG2 cells were transfected with a vector or plasmids expressing human CD320 WT and the mutant QQQ. The transfected cells cultured without or with Ben-gal (BG) were incubated with a medium containing vitamin B_12_. After 24 h at 37 °C, the cells were lysed by sonication. Vitamin B_12_ levels in the lysates were measured by ELISA. Data are mean ± SD (n = 4) analyzed by one-way ANOVA.

CD320-mediated vitamin B_12_ uptake has been shown in cultured HepG2 cells (46). To assess the importance of CD320 N- and O-glycosylation in vitamin B_12_ uptake, we transfected HepG2 cells with a vector (control) or plasmids expressing CD320 WT and the mutant QQQ lacking the N-glycosylation sites. The cells were cultured in a DMEM medium with vitamin B_12_ in the absence or presence of Ben-gal. After 24 h, the cells were lysed and vitamin B_12_ levels were quantified by ELISA. Similarly high levels of vitamin B_12_ were detected in lysates from the cells expressing CD320 WT and the mutant QQQ, compared to those in the vector-transfected cells (Fig. 8). In lysates from CD320 WT-expressing cells treated with Ben-gal, vitamin B_12_ levels were markedly decreased (Fig. 8). These results were consistent, indicating that N-glycosylation in CD320 was unnecessary for vitamin B_12_ uptake, whereas inhibition of O-glycosylation in CD320 decreased vitamin B_12_ uptake in HepG2 cells.

### Importance of the Ser/Thr-rich region for CD320 cell surface expression

**Figure 9:**
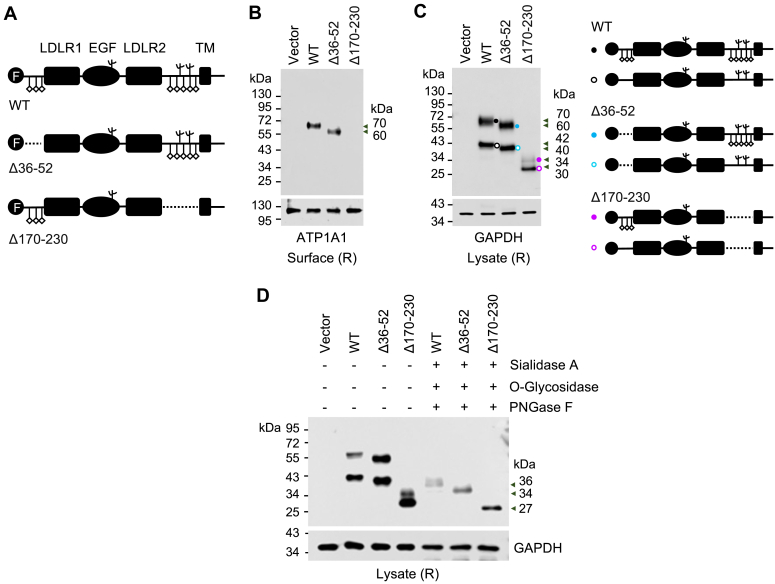
**Analysis of clustered O-glycosylation sites in human CD320.***A*, predicted O-glycosylation sites (vertical lines with a diamond) cluster near the N-terminus (residues 36–52) and the Ser/Thr-rich region near the transmembrane (TM) domain (residues 170–230) of human CD320. Three N-glycosylation sites (Y-shaped symbols) are indicated. F: FLAG tag. Plasmids expressing the CD320 mutants lacking residues 36 to 52 (Δ36–52) or 170 to 230 (Δ170–23) were made. *B* and *C*, western blotting of CD320 proteoforms among biotin-labeled surface proteins (*B*) and in lysates (*C*) from transfected HEK293 cells. Western blotting was done using an anti-FLAG antibody under reducing conditions (R). ATP1A1 (*B*) and GAPDH (*C*) were controls for protein loading. In *C*, bands on the western blot likely represented proteoforms of CD320 WT and the mutants with or without N- and O-glycans. *D*, CD320 WT and the mutants were expressed in transfected HEK293 cells. Cell lysates were treated without (−) or with (+) sialidase A, O-glycosidase, and PNGase F. Data are representative of three experiments.

As indicated by bioinformatics analysis (Table S1), the potential O-glycosylation sites in CD320 are located mostly at the N-terminus (residues 36–52) and the Ser/Thr-rich region near the transmembrane domain (residues 170–230) (Fig. 9*A*). To test the importance of these two regions for CD320 cell surface expression, we made plasmids encoding CD320 Δ36 to 52 and Δ170 to 230 mutants, in which residues 36 to 52 and 170 to 230 were deleted, respectively (Fig. 9*A*). Based on AlphaFold three software analysis, the deletion of these separate sequences would not disrupt the overall folding of the major LDLR1-EGF-LDLR2 segment (Fig. S4). We expressed these mutants in HEK293 cells and analyzed their expression on the cell surface. In western blotting, CD320 WT and the mutant Δ36 to 52, but not the mutant Δ170 to 230, were detected among biotin-labeled cell surface proteins (Fig. 9*B*). In western blotting of lysates from the transfected cells, CD320 bands of WT (∼70 and ∼42 kDa), the mutant Δ36 to 52 (∼60 and ∼40 kDa), and the mutant Δ170 to 230 (∼34 and ∼30 kDa) were detected, which likely represented N- and/or O-glycosylated proteoforms of these proteins (Fig. 9*C*). Consistently, when the lysates were treated with sialidase A, O-glycosidase, and PNGase F, single bands of ∼36, ∼34, and ∼27 kDa were detected in samples from WT and mutants Δ36 to 52 and Δ170 to 230, respectively (Fig. 9*D*). These results indicated that O-glycosylation in the Ser/Thr-rich region near the transmembrane domain was important for CD320 expression on the cell surface.

## Discussion

CD320 is essential in vitamin B_12_ metabolism. To date, posttranslational modifications in human CD320 have not been fully elucidated. Previously, a soluble receptor of ∼58 kDa from human placental membrane preparations, which bound to holoTC, was shown to contain ∼17 kDa of carbohydrates (47). Based on glycosidase digestion and lectin binding, the predicted carbohydrates on the receptor included sialic acids, N-glycans, and O-glycans (47). At the time of the study, the molecular identity of the soluble receptor was not established. Possibly, the placental membrane-derived soluble receptor was a cleaved extracellular fragment of CD320.

In this study, we analyzed human CD320 in HEK293 and HepG2 cells. We showed that in the cytoplasm CD320 existed in two major forms: an ∼42-kDa band and an ∼70-kDa band. Most likely, the ∼42-kDa band represented N-glycosylated CD320 in the ER, whereas the ∼70-kDa band represented the CD320 proteoform undergone N- and O-glycosylation in the ER and the Golgi. In line with this hypothesis, treatment with sialidase A, O-glycosidase, and PNGase F reduced the ∼42- and ∼70-kDa bands to ∼36 kDa. The theoretical molecular mass of the recombinant human CD320 with a FLAG tag was ∼29 kDa, smaller than the ∼36-kDa band observed on western blots. Further studies are required to determine if CD320 underwent additional posttranslational modifications that may explain the apparent discrepancy between the theoretical and observed molecular masses in our study.

In western blotting of biotin-labeled proteins under reducing conditions, we found that the ∼70-kDa, but not the ∼42-kDa, CD320 band was on the cell surface. However, the ∼70-kDa band was absent on the cell surface when the blotting was done under non-reducing conditions. Instead, CD320 appeared as a band of ∼350 kDa. By analyzing CD320 proteins with separate tags in immunoprecipitation and western blotting, we found that the ∼350-kDa band was likely a CD320 homopolymer, probably a pentamer. Additional studies are required to determine the structure of the ∼350-kDa CD320 complex.

Calnexin is a chaperone that mediates glycoprotein folding in the ER (48, 49). The function of calnexin depends on its interaction with N-glycans on the client proteins. In many transmembrane proteases, including corin and hepsin (25, 26, 50), abolishing N-glycosylation sites causes protein misfolding and retention in the ER. In mammals ranging from mice to humans, N-glycosylation sites in CD320 proteins are conserved, an indication of functional importance. In this study, we found that all three predicted N-glycosylation sites in human CD320 were glycosylated. However, N-glycosylation was dispensable for CD320 cell surface expression. Apparently, N-glycans were not required for CD320 folding in the ER and subsequent intracellular trafficking. Moreover, we found that vitamin B_12_ uptakes were similar in cells expressing CD320 with (*i.e.*, WT) and without (*i.e.*, the QQQ mutant) N-glycosylation. A previous study also reported that N-glycosylation sites were unnecessary for holoTC binding (22). Additional studies will be important to understand the functional significance of N-glycans in CD320.

O-glycans are important in protein trafficking and cell membrane targeting (40, 51, 52, 53, 54). For example, defective O-glycosylation in decay accelerating factor (DAF), a glycosylphosphatidylinositol (GPI)-anchored membrane protein, decreased DAF expression on the cell surface, due to increased proteolytic cleavage (55). In colorectal HT-29 cells, inhibition of O-glycosylation by Ben-gal blocked the intracellular trafficking of membrane-bound glycoproteins, including dipeptidyl peptidase-4 (a GPI-anchored protein) and MUC1 (a membrane-type mucin) (45). We also found that Ben-gal treatment inhibited O-glycosylation and cell surface expression of CD320 in HEK293 and HepG2 cells. Consistently, vitamin B_12_ uptake was markedly decreased in the Ben-gal-treated HepG2 cells. By contrast, similar Ben-gal treatment did not block corin (without O-glycans) and thrombomodulin (with O-glycans) expression on the cell surface, indicating that the observed effect of Ben-gal on the cell surface expression of CD320 was not due to major disruption of intracellular trafficking of membrane proteins. In the lectin binding experiments with MAL-II and PNA, we found that Ben-gal treatment inhibited sialylation of O-glycans on CD320, which blocked PNA binding. In line with our findings, sialic acid removal from tumor-associated antigens has been shown to enhance PNA binding (56). Together, these findings highlight the crucial role of O-glycans in CD320 intracellular trafficking and cell membrane expression, which is important for vitamin B_12_ uptake.

Most O-glycosylation happens in the Golgi. In Notch signaling proteins, O-fucosylation in EGF repeats occurs in the ER, which regulates protein folding, stability, and trafficking (57, 58). CD320 has an EGF-like domain (21). In the cells treated with monensin disrupting the Golgi network, levels of the ∼42-kDa CD320 band remained unchanged (Fig. 2*C*), indicating that this CD320 proteoform was in the ER, but not the Golgi. As shown with glycosidase digestion, the ∼42-kDa proteoform contained N-glycans but no detectable O-glycans (Figs. 3 and 5, *A* and *C*), indicating that in HEK293 cells O-glycosylation in CD320 occurred in the Golgi. When the cells were treated with BFA, the ∼42-kDa band was eliminated (Fig. 5*B*), whereas the level of the ∼42-kDa band was increased in the Ben-gal-treated cells (Fig. 6, *A* and *B*). BFA inhibits protein trafficking from the ER to the Golgi, but not from the Golgi to the ER (33, 34). In the BFA-treated cells, glycosyltransferases in the Golgi are redistributed to the ER (36). This could cause CD320 O-glycosylation in the ER, explaining the lack of the ∼42-kDa band in the BFA-treated cells. On the other hand, Ben-gal treatment inhibited the intracellular trafficking of CD320. The high levels of the ∼42-kDa band in the Ben-gal-treated cells indicated that reduced CD320 trafficking in the Golgi may cause CD320 accumulation in the ER. By testing the Endo H sensitivity of CD320 proteoforms from the cells without or with Ben-gal treatment, we found no evidence that the Ben-gal treatment might cause the retrograde transport of CD320 under our experimental settings.

Many cell surface receptors with LDLR and/or EGF repeats, *e.g.*, the LDLR and ApoE receptor-2, have an O-glycosylated Ser/Thr-rich region near the transmembrane domain, which is functionally important (59, 60). In human CD320, the predicted O-glycosylation sites cluster in two areas: residues 36 to 52 at the N-terminus and residues 170 to 230 in the Ser/Thr-rich region near the transmembrane domain. Deletion of residues 170 to 230, but not 36 to 52, blocked CD320 expression on the surface of HEK293 cells, indicating that O-glycosylation in the Ser/Thr-rich region was critical for CD320 intracellular trafficking and cell surface expression. Remarkably, the autoantibodies targeting CD320 identified in patients with neurologic deficits have been found to recognize a specific epitope (residues 183–197) in the same region (14). These findings highlight the importance of the Ser/Thr-rich region in CD320 function and associated pathology.

Currently, the specific O-glycan types on CD320 and the molecular mechanism underlying the O-glycan function in CD320 trafficking remain unknown. In some membrane-associated proteins, O-glycans participate in an apical membrane targeting mechanism, which acts beyond the *cis*-Golgi in polarized epithelial cells (45, 61). CD320 is also expressed on the apical membrane in polarized renal and intestinal epithelial cells (18, 23). Previously, we identified a protein motif in the second LDLR domain of CD320 that acted as an apical sorting signal in a Rab11a-mediated mechanism (18, 23). In principle, the O-glycans on CD320 may be part of the membrane sorting mechanism or act as an independent mechanism in protein trafficking in the Golgi. More studies, *e.g.*, mass-spectrometry and proteomic analyses, shall help to define specific positions and the extent of O-glycosylation in CD320 and to elucidate the mechanism underlying the role of O-glycans in CD320 biology.

In summary, CD320 is a key receptor in vitamin B_12_ metabolism. Here, we show that CD320 undergoes N- and O-glycosylation and sialylation before forming a high-molecular-weight complex on the cell surface. In transfected HEK293 and HepG2 cells, abolishing N-glycosylation sites does not block CD320 intracellular trafficking and expression on the cell surface. In contrast, treatment of the cells with Ben-gal, a structural analog of GalNAc-α-1-O-Ser/Thr, inhibits O-glycosylation and cell surface expression of CD320, thereby preventing vitamin B_12_ uptake. Studies with CD320 deletion mutants indicate that O-glycosylation in the Ser/Thr-rich region near the transmembrane domain is critical for the cell surface expression of CD320. These findings provide important new insights into the cellular mechanisms that regulate CD320 expression and function.

## Experimental procedures

### Plasmids

Plasmid encoding human WT CD320 was reported previously (21). Site-directed mutagenesis (ClonExpress One Step Cloning, Vazyme, C112) was done to make plasmids expressing CD320 mutants, including N126Q, N195Q, N213Q, N126Q/N195Q, N126Q/N213Q, N195Q/N213Q, and N126Q/N195Q/N213Q, in which the predicted N-glycosylation sites were mutated individually or in combination, and the deletion mutants Δ36 to 52 and Δ170 to 230. All CD320 proteins expressed by these plasmids had an N-terminal FLAG tag. To examine CD320 complex formation, we made another plasmid expressing CD320 with an N-terminal V5 tag. The plasmid expressing human transmembrane protease corin with a C-terminal V5 tag was published previously (62). The plasmid expressing human thrombomodulin with an N-terminal FLAG tag was made using a previously published plasmid as a template (63).

### Prediction of O-glycosylation sites on CD320 and thrombomodulin

The human CD320 and thrombomodulin protein sequences (NCBI accession numbers: NP_057663.1 and NP_000352) were analyzed by the bioinformatic software (NetOGlyc 4.0) for potential O-glycosylation sites (https://services.healthtech.dtu.dk/services/NetOGlyc-4.0/). The predicted O-glycosylation sites in human CD320 and thrombomodulin are listed in Tables S1 and S2, respectively.

### Cell culture and transfection

HEK293 (ATCC, CRL-1573, authenticated by short tandem repeat (STR) profiling) and HepG2 (ATCC, HB-8065, authenticated by STR profiling) cells were cultured in Dulbecco’s modified Eagle’s medium (DMEM) (Corning, 10–0130CVRC) with 10% fetal bovine serum (FBS) (Gibco, 16000–044) in an incubator at 37 °C with 5% CO_2_ (27). After the cells reached 80% confluency, expression plasmids or a control vector were transfected into cells using PolyJet In Vitro DNA Transfection Reagent (SignaGen Laboratories, SL100688). After 6 h, the cells were switched to fresh medium and cultured for an additional 24 to 48 h before being used for protein analysis.

### Western blotting

The transfected cells were washed with phosphate-buffered saline (PBS) and lysed with 1% Triton X-100 in a Tris-HCI solution, pH 8.0, with 150 mM NaCl and a mixture of protease inhibitors (1:100, Roche Applied Science, 04693116001). Proteins in the lysates were quantified by a bicinchoninic acid (BCA)-based assay. The samples were diluted in Laemmli buffer (Bio-Rad, 161–0737) with (reducing) or without (non-reducing) β-mercaptoethanol (2.5% v/v), separated by SDS-PAGE, and transferred onto a polyvinylidene difluoride membrane. Western blotting was done using horseradish peroxidase (HRP)-conjugated antibodies against FLAG (1:10,000, Sigma, A8592) or V5 (1:5000, Thermo Fisher, R96125) tags. As controls for protein sample loading, western blots were re-probed with an antibody against glyceraldehyde-3-phosphate dehydrogenase (GAPDH) (1:10,000, Bioworld, MB001H) for proteins in cell lysates. After washing, the western membranes were treated with an electrochemiluminescent solution (NCM Biotech, P10050) and exposed to an analyzer (Amersham Imager 600).

### Analysis of cell surface proteins

To label cell surface proteins, the transfected cells were treated with Sulfo-NHS-SS-biotin (0.25 mg/ml) (Thermo Fisher, 89,881) on ice. After 4 min, a glycine solution (100 mM) was added to terminate the reaction. After 15 min on ice, the cells were lysed. Biotin-labeled proteins were precipitated with NeutrAvidin beads (Thermo Fisher, 89881) and analyzed by western blotting with an anti-FLAG antibody (1:2000, Sigma, F1804). As a control for cell membrane proteins, western blots were re-probed with an antibody against Na^+^/K^+^ ATPase 1 (ATP1A1) (1:1000, ZenBio, R380790) and an HRP-conjugated secondary antibody (1:10,000, Boster Bio, BA1054). To analyze CD320 bands in the conditioned media, immunoprecipitation was done with the anti-FLAG antibody at 4 °C. After 16 h, protein A-Sepharose beads (Thermo Fisher, 101042) were added to pull down the antibody-coupled proteins for SDS-PAGE and western blotting analysis.

To verify proteins on the cell surface, the transfected cells expressing CD320 were treated with 0.05% Trypsin-EDTA (Gibco, 25300) on ice. After 1 to 15 min, DMEM with 10% FBS was added to inhibit trypsin activity. The detached cells were collected, washed once with PBS, and lysed as described earlier. Proteins in the lysates were analyzed by SDS-PAGE and western blotting.

### Glycosidase digestion

To analyze N- and O-glycosylation on CD320, lysates from the transfected cells were treated with glycosideases, including PNGase F from *Flavobacterium meningosepticum* (NEB, P0704S) to remove almost N-linked oligosaccharides, Endo H from *Streptomyces picatus* (NEB, P0702S) to cleave the chitobiose core of high mannose and some hybrid oligosaccharides, sialidase A from *Arthrobacter ureafaciens* (NEB, P0722S) to remove branched sialic acid residues, and O-glycosidase from *Enterococcus faecalis* (NEB, P0733S) to remove Core one and Core 3 O-linked disaccharides. The lysates (10 μg) were diluted in denaturing buffer, boiled for 10 min, and treated with the glycosidases at 37 °C followed by SDS-PAGE and western blotting.

### Monensin, BFA, and Ben-gal treatments

The transfected HEK293 cells were treated with different concentrations of monensin (0.05–0.2 μM) (Sigma, 475897), BFA (0.1–1 μM) (Sigma, 203729) in dimethylsulfoxide (DMSO), or Ben-gal (8 mM) (Sigma, B4894) in DMEM. In controls, the cells were treated with vehicles (DMSO or DMEM). After 24 h at 37 °C, the cells were washed once with PBS and lysed, as described above. CD320 bands in the lysates were analyzed by SDS-PAGE and western blotting, as described above.

### Lectin-binding experiments

HEK293 cells were transfected with the plasmid expressing WT CD320 or a control vector. The cells were cultured at 37 °C in the presence of Ben-gal, as described above. After 24 h, the cells were lysed and CD320 proteoforms were precipitated with the anti-FLAG antibody and protein A beads. The precipitated proteins were analyzed by SDS-PAGE and western blotting. The western blots were also incubated with biotinylated MAL-II (0.1 μg/ml) (VectorLabs, B-1265-1) and HRP-conjugated streptavidin (1:1000, Beyotime, A0305) or HRP-conjugate PNA (0.2 μg/ml) (Sigma-Aldrich, L7759) at room temperature for 2 h. The lectin-bound bands were detected with the chemiluminescent substrate.

### Measurement of vitamin B_12_ by ELISA

HepG2 cells transfected with a vector or CD320-expressing plasmid were cultured in DMEM medium with 2% fetal bovine serum and 500 nM vitamin B_12_ (MedChemExpress, HY-B0315) without or with Ben-gal (16 mM). After 24 h at 37 °C, the cells were suspended and lysed by sonication. Proteins in the lysates were quantified by the BCA assay. Vitamin B_12_ levels were measured by an ELISA kit (Shanghai Yansheng Industrial, YS-S964804) according to the manufacturer’s instructions.

### Protein three-dimensional (3D) model prediction

The artificial intelligence based AlphaFold three software was used to predict 3D structures of human CD320 variants. Protein sequences of human CD320 WT (NCBI accession number: NP_057663.1) and the deletion mutants lacking residues 36 to 52 (Δ36–52) or 170 to 230 (Δ170–230) were submitted to the AlphaFold three website (https://www.alphafoldserver.com). Protein 3D models were generated with corresponding pLDDT (predicted Local Distance Difference Test) values to indicate the potential accuracy of amino acid positions in local structures.

### Statistical analyses

Data were analyzed with Prism 9.0 software (Graphpad). Data normal distribution of was verified using the Shapiro-Wilk test. Student’s *t* test was used to compare data between two groups and one-way ANOVA followed by Bonferroni’s *post hoc* analysis was used to compare data from three or more groups. A *p* value of <0.05 is considered significant. Data are presented as mean ± SD or SEM, as indicated.

## Data availability

All primary data are available upon request.

## Supporting information

This article contains supporting information.

## Conflict of interest

The authors declare no conflict of interest with the contents of this article.
