# Supplementary material for: O-glycosylation is essential for cell surface expression of the transcobalamin receptor CD320
Source: J Biol Chem. 2024 Nov 16;300(12):107997. doi: 10.1016/j.jbc.2024.107997 (PMC11667166; doi:10.1016/j.jbc.2024.107997)
Supplement: Supplementary Figures [file mmc1.pdf]

Du et al. Supplementary Figures 1-4.

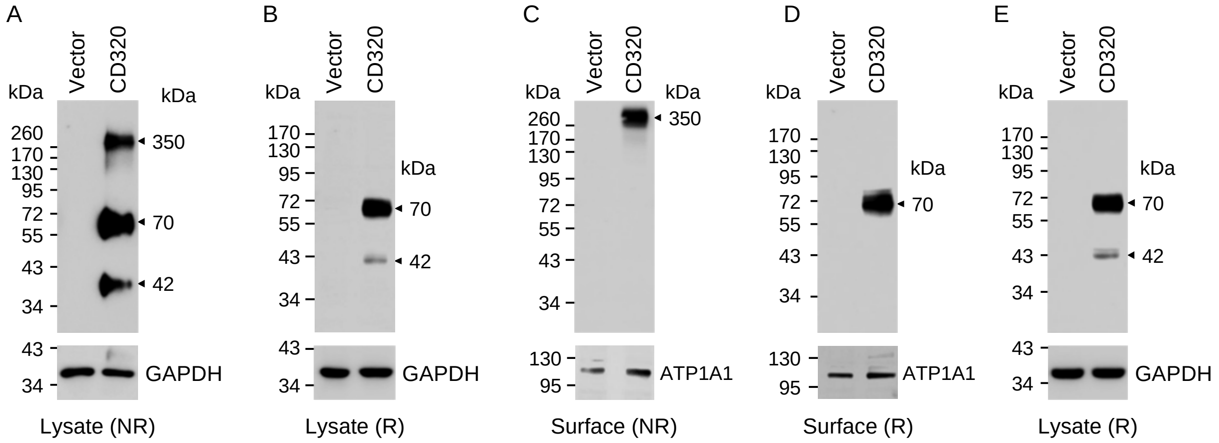

Figure S1. Western blotting of human CD320 proteoforms in HepG2 cells. *A* and *B*, HepG2 cells were transfected with a vector or CD320-expressing plasmid. CD320 proteoforms were analyzed by western blotting under non-reducing (NR) (*A*) and reducing (R) (*B*) conditions. GAPDH was a protein loading control. *C*-*E*, Western blotting of corin CD320 proteoforms in biotin-labeled cell surface proteins under NR (*C*) and R (*D*) conditions. ATP1A1 was used as a control for membrane proteins. CD320 protein expression in cell lysates from the same samples were verified by western blotting under R conditions (*E*). Data are representative of three experiments.

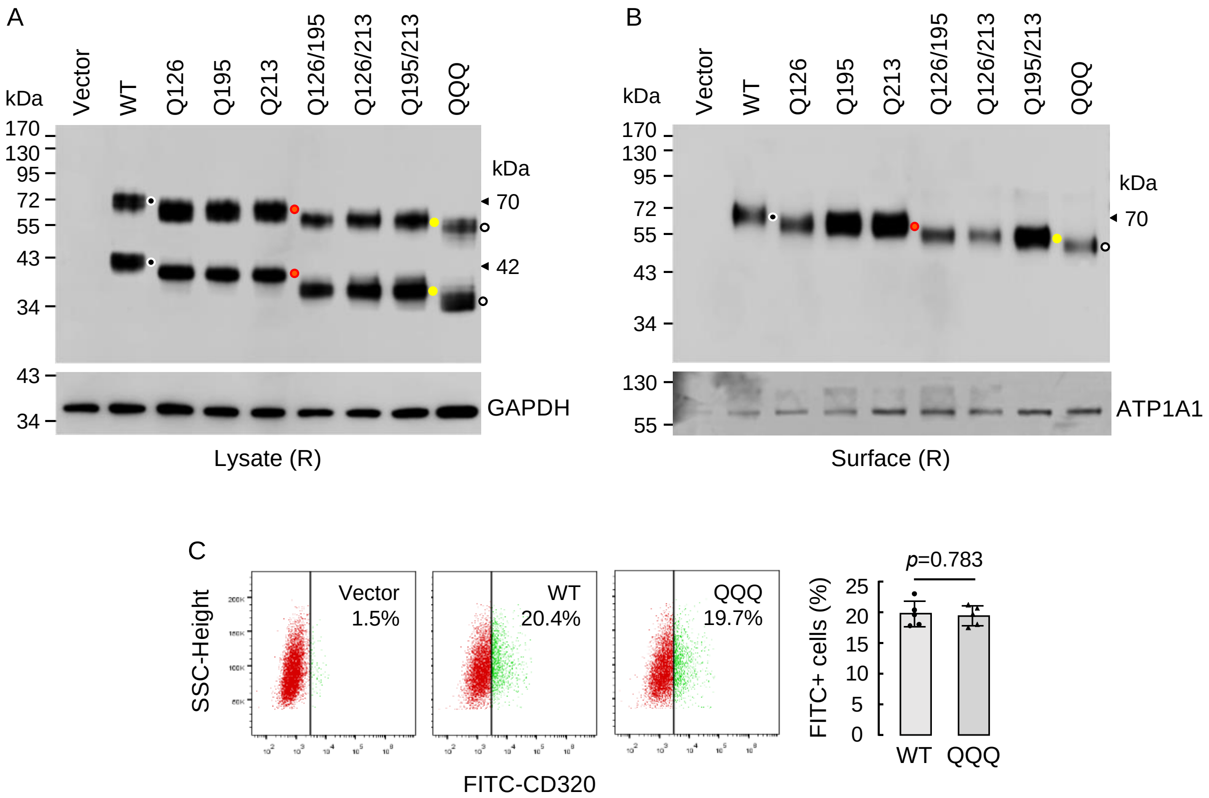

Figure S2. Effects of N-glycosylation at the predicted sites on CD320 expression in HepG2 cells. *A*, Western blotting of CD320 proteoforms in lysates from transfected HepG2 cells under reducing conditions (R). CD320 WT appeared as two bands of ~70 and ~42 kDa, respectively (black dots). The mobility of these bands increased progressively in the mutants lacking one (red dots), two (yellow dots), or three (white dots) N-glycosylation sites. GAPDH was a protein loading control. *B*, Western blotting of CD320 proteoforms in biotin-labeled surface proteins from HepG2 cells expressing CD320 WT (black dot) and the mutants lacking one (red dot), two (yellow dot), or three (white dot) N-glycosylation sites. ATP1A1 was a control for membrane proteins. *C*, Flow cytometric analysis of CD320-positive HepG2 cells transfected with plasmids expressing CD320 WT and the mutant QQQ or a control vector. Data in (*A* and *B*) are representative from three experiments. Data in (*C*) (n = 5) were analyzed by Student's *t* test.

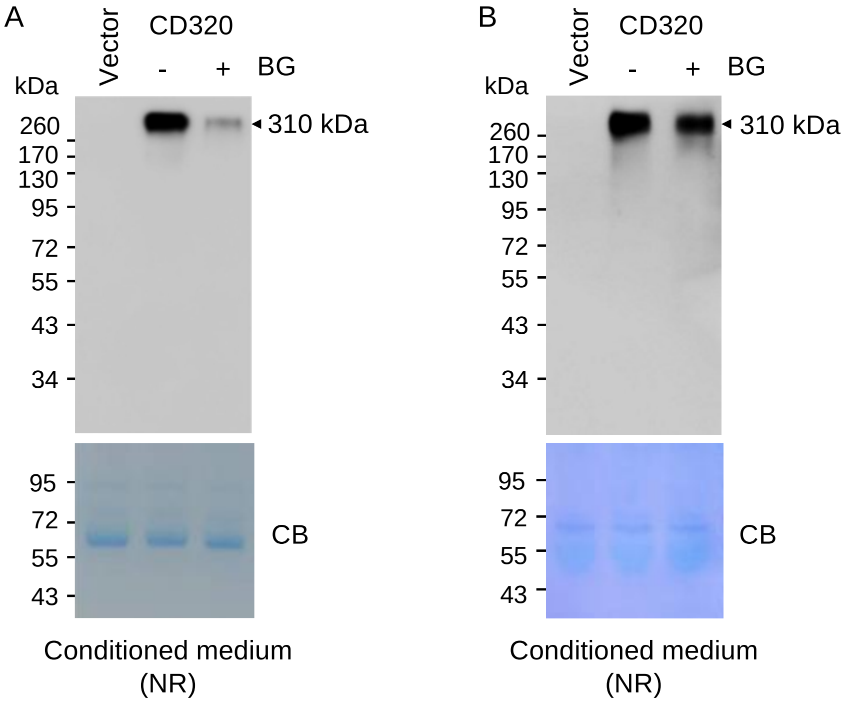

Figure S3. Analysis of CD320 proteoforms in the conditioned media. *A* and *B*, HEK293 (*A*) and HepG2 (*B*) cells were transfected with a vector or plasmid expressing WT CD320. The cells were cultured without (-) or with (+) Ben-gal (BG) (8 mM) at 37°C for 24 h. The conditioned media were collected and CD320 was immunoprecipitated and analyzed by western blotting under non-reducing (NR) conditions. Coomassie blue (CB)-stained non-specific bands in the conditioned media were used as a control for protein loading.

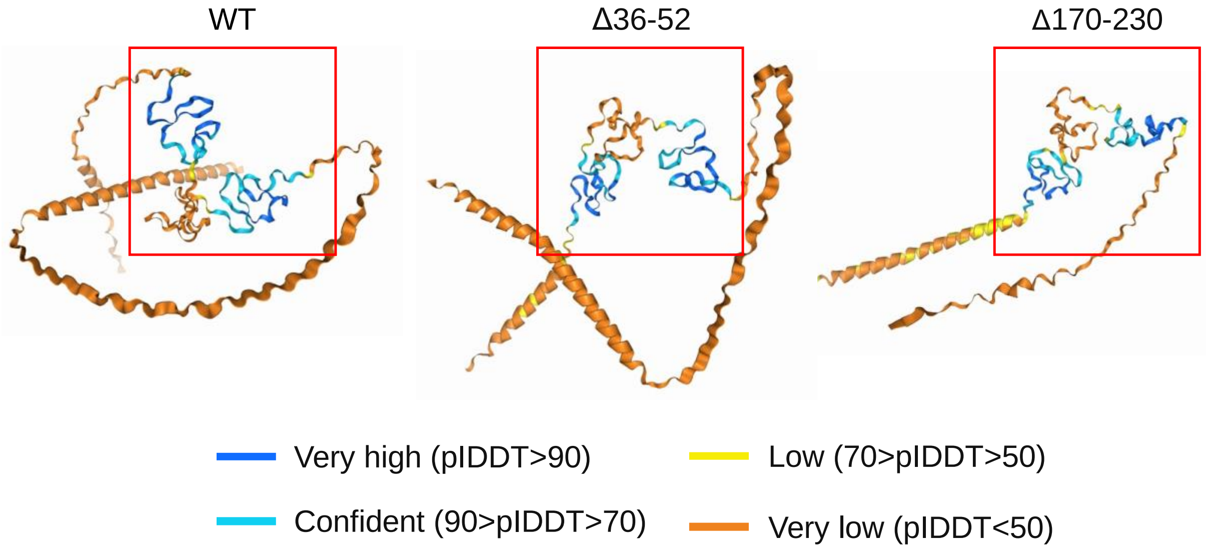

Figure S4. Predicted 3D models of human CD320 WT and deletion mutants. AlphaFold 3 software was used to predict 3D structures of human CD320 WT (*left*), the mutant Δ36-52 (*middle*), and the mutant Δ170-230 (*right*). Protein sequences of human CD320 WT (NCBI accession number: NP_057663.1) and the deletion mutants lacking residues 36-52 (Δ36-52) or 170-230 (Δ170-230) were submitted to the AlphaFold 3 website (https://www.alphafoldserver.com). Protein 3D models were generated with corresponding pLDDT (predicted Local Distance Difference Test) values to indicate the potential accuracy of amino acid positions in local structures. The LDLR1-EGF-LDLR2 segments in CD320 WT and the mutants are indicated by red boxes.
