# Supplementary material for: O-glycosylation is essential for cell surface expression of the transcobalamin receptor CD320
Source: J Biol Chem. 2024 Nov 16;300(12):107997. doi: 10.1016/j.jbc.2024.107997 (PMC11667166; doi:10.1016/j.jbc.2024.107997)
Supplement: Supplementary Tables [file mmc2.pdf]

Du et al. Supplemental Tables.

Table S1. The predicted O-glycosylation sites in human CD320.

Domain	Amino acid	Position	Score
N-terminal	S	36	0.50740
	S	39	0.59136
	T	40	0.55821
	T	42	0.67129
	S	43	0.84656
	S	50	0.71523
	S	51	0.55047
LDLR1	S	53	0.76931
Between LDLR2 and TM	T	180	0.56388
	T	186	0.60955
	S	189	0.59403
	S	192	0.64936
	T	197	0.70752
	T	198	0.88607
	T	204	0.74234
	S	207	0.60766
	S	210	0.58948
	T	215	0.50000
	S	216	0.66716
	S	217	0.77923
	S	218	0.50000
	S	223	0.85633
	T	227	0.54766

TM: transmembrane. NetOGlyc-4.0 analysis was last verified on June 13, 2024.

Table S2. The predicted O-glycosylation sites in human thrombomodulin.

Domain	Amino acid	Position	Score
C-type lectin-like domain (CTLD)	T	112	0.74474
	S	118	0.72484
	S	120	0.77069
	S	141	0.88683
	T	146	0.62614
	S	149	0.91232
Between CTLD and EGF1	T	174	0.58199
	S	190	0.68046
	T	192	0.80233
	T	195	0.76389
	S	211	0.54055
	S	212	0.66215
	T	225	0.68942
EGF1	S	279	0.69036
	T	281	0.66255
Between EGF1 and 2	S	283	0.78375
EGF2	T	285	0.68877
	S	287	0.68878
EGF6	T	478	0.52104
Between EGF6 and TM	S	490	0.72632
	S	498	0.87927
	T	500	0.66092
	S	503	0.61073
	T	504	0.85566
	T	506	0.64346

TM: transmembrane. NetOGlyc-4.0 analysis was last verified on June 13, 2024.
